# Aurora A, MCAK, and Kif18b promote Eg5-independent spindle formation

**DOI:** 10.1007/s00412-016-0607-4

**Published:** 2016-06-29

**Authors:** Roy G. H. P. van Heesbeen, Jonne A. Raaijmakers, Marvin E. Tanenbaum, Vincentius A. Halim, Daphne Lelieveld, Cor Lieftink, Albert J. R. Heck, David A. Egan, René H. Medema

**Affiliations:** 1 Division of Cell Biology, The Netherlands Cancer Institute, Amsterdam, The Netherlands; 2 Hubrecht Institute, The Royal Netherlands Academy of Arts and Sciences (KNAW) and University Medical Center Utrecht, Utrecht, the Netherlands; 3 Biomolecular Mass Spectrometry and Proteomics Group, Bijvoet Center for Biomolecular Research and Utrecht Institute for Pharmaceutical Sciences, Utrecht University, Utrecht, The Netherlands; 4 Cell Screening Core, Department of Cell Biology, Center for Molecular Medicine, University Medical Centre, Utrecht, The Netherlands; 5 Division of Molecular Carcinogenesis, The Netherlands Cancer Institute, Amsterdam, The Netherlands

**Keywords:** Eg5, Kif15, Aurora A, Kif18B, MCAK, Spindle

## Abstract

Inhibition of the microtubule (MT) motor protein Eg5 results in a mitotic arrest due to the formation of monopolar spindles, making Eg5 an attractive target for anti-cancer therapies. However, Eg5-independent pathways for bipolar spindle formation exist, which might promote resistance to treatment with Eg5 inhibitors. To identify essential components for Eg5-independent bipolar spindle formation, we performed a genome-wide siRNA screen in Eg5-independent cells (EICs). We find that the kinase Aurora A and two kinesins, MCAK and Kif18b, are essential for bipolar spindle assembly in EICs and in cells with reduced Eg5 activity. Aurora A promotes bipolar spindle assembly by phosphorylating Kif15, hereby promoting Kif15 localization to the spindle. In turn, MCAK and Kif18b promote bipolar spindle assembly by destabilizing the astral MTs. One attractive way to interpret our data is that, in the absence of MCAK and Kif18b, excessive astral MTs generate inward pushing forces on centrosomes at the cortex that inhibit centrosome separation. Together, these data suggest a novel function for astral MTs in force generation on spindle poles and how proteins involved in regulating microtubule length can contribute to bipolar spindle assembly.

**Electronic supplementary material:**

The online version of this article (doi:10.1007/s00412-016-0607-4) contains supplementary material, which is available to authorized users.

## Introduction

The bipolar spindle is a microtubule (MT)-based structure required for successful chromosome segregation during mitosis. Assembly of the bipolar spindle requires tight regulation of a wide variety of microtubule-associated proteins (MAPs), including MT motors from the kinesin family of proteins (Walczak and Heald 2008). An essential and highly conserved protein for bipolar spindle assembly is kinesin-5 (Eg5 in humans). Eg5 forms a unique tetrameric configuration, hereby enabling it to crosslink and slide antiparallel MTs apart and thereby driving centrosome separation and bipolar spindle assembly (Kashina et al. 1996; Kapitein et al. 2005). Inhibition or depletion of Eg5 results in a mitotic arrest and subsequent cell death due to the formation of monopolar spindles in nearly all organisms tested (Sawin et al. 1992; Blangy et al. 1995; Mayer et al. 1999; Ferenz et al. 2010). Therefore, Eg5 is an attractive anti-mitotic target for cancer therapy (Rath and Kozielski 2012).

Recent studies reported the existence of redundant pathways, cooperating with Eg5 to drive centrosome separation and bipolar spindle assembly. In human cells, kinesin-12 (Kif15/Hklp2 in humans) was identified to cooperate with Eg5 in bipolar spindle assembly (Tanenbaum et al. 2009; Vanneste et al. 2009). Ectopic overexpression of Kif15 bypasses the requirement for Eg5 in bipolar spindle assembly (Tanenbaum et al. 2009). In addition, we and others have shown that human cells, treated with Eg5 inhibitors can easily acquire the ability to build a bipolar spindle in the absence of Eg5 activity, but become dependent on Kif15 for bipolar spindle formation (Raaijmakers et al. 2012; van Heesbeen et al. 2013; Sturgill and Ohi 2013; Ma et al. 2014; Sturgill et al. 2016).

To identify genes that are required for Eg5-independent bipolar spindle assembly, we performed a genome-wide small interfering RNA (siRNA) screen in HeLa and HeLa-derived Eg5-independent cells (EICs, (Raaijmakers et al. 2012). We searched for genes that specifically arrested EICs in mitosis, using a high content, fixed cell assay. We identified the mitotic kinase Aurora A and two kinesins that regulate MT dynamics, MCAK (Kif2C/kinesin-13) and Kif18b (kinesin-8), to be essential for bipolar spindle assembly in EICs. Our data reveals two novel mechanisms that are required for Eg5-independent bipolar spindle assembly and uncovers three potential targets for combination therapy with Eg5 inhibitors.

## Results

### A genome-wide siRNA screen identifies three genes required for bipolar spindle assembly in Eg5-independent cells

**Fig. 1:**
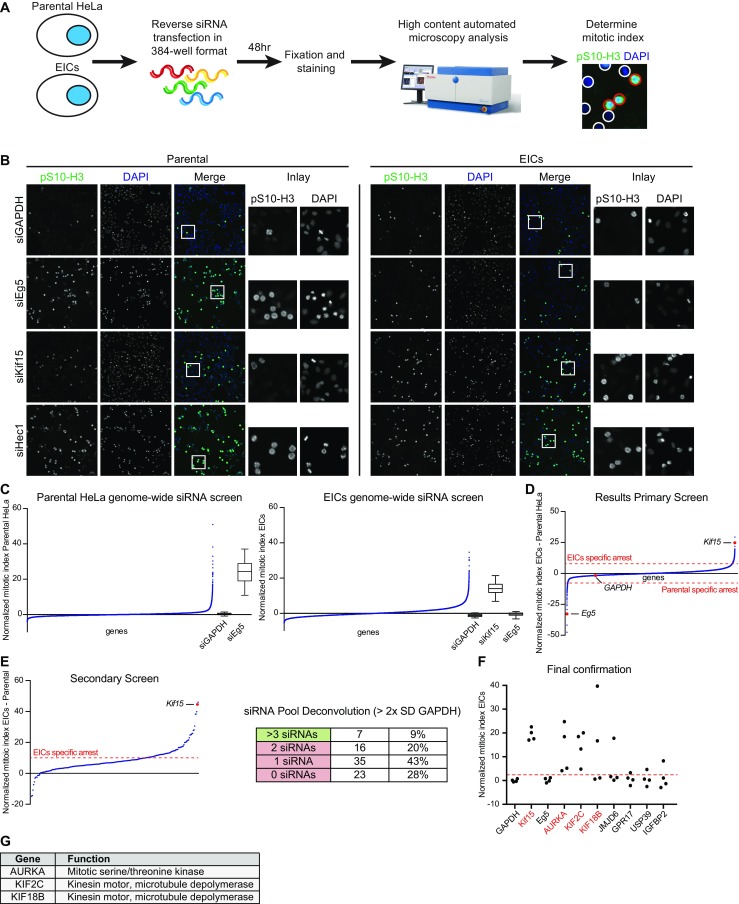
Genome-wide siRNA screen identifies three genes specifically affecting mitosis in Eg5-independent cells. **a** Experimental setup of the screen. Parental and EICs HeLa cells were reverse transfected in a 384-well format with a genome wide ON-Target plus siRNA library from Dharmacon. Forty-eight hours after transfection, the cells were fixed and mitotic cells were stained using the marker phospho-histone H3. The mitotic index was determined using an automated fluorescence microscope. **b** Representative pictures of parental cells and EICs, treated with the indicated siRNAs. GAPDH and Hec1 siRNA served as non-specific negative and positive controls, respectively. Eg5 siRNA served as a parental-specific control and Kif15 as a EICs-specific control. **c** Results from the primary screen. The *left graph* and *middle graph* show the normalized mitotic indexes, ordered from lowest to highest for the parental cells and the EICs, respectively. *Box plots* on the right site of the graphs show the controls for the indicated cell line. Note that depletion of Eg5 shows a high mitotic index in the parental cells, while it has no significant effect in the EICs. Kif15 served as a EICs specific positive control. **d** The *graphs* show the normalized mitotic index after subtraction of parental screen scores from the EICs screen scores. Note that as expected, Eg5 and Kif15 were found as clear outliers. Genes above the *upper red-dotted line* indicate an EICs-specific mitotic arrest, genes below the *lower red-dotted line* show a parental specific mitotic arrest. **e** Results from the secondary screen, after subtraction of the normalized mitotic of the parental cells from the scores of the EICs. The 85 genes above the *red-dotted line* were selected for siRNA deconvolution. The *right table* shows the results from the siRNA deconvolution. Seven genes from the original 85 were confirmed on-target and selected for final confirmation. **f, g** The final confirmation experiment identified three hits to be specific for the EICs

In order to identify genes contributing to centrosome separation and bipolar spindle assembly in EICs, we performed a high content, image-based genome-wide siRNA screen in these cells. We selected an EIC clone that did not overexpress Kif15 and neither contained mutations in Eg5, two mechanisms that were previously described to promote Eg5 inhibitor resistance (Wacker et al. 2012; Raaijmakers et al. 2012; Sturgill et al. 2016). The selected clone was previously described to grow completely independent of Eg5-activity as siRNA-mediated depletion of Eg5 did not affect proliferation of these cells (clone 1 from (Raaijmakers et al. 2012)). A schematic depiction of the experimental setup of the screen is shown in Fig. 1a. In short, cells were transfected with pools of ON-TARGET plus siRNAs containing four duplexes per gene, targeting 18,104 human genes in total in a 384-well format (approximately 80 % of the human genome, see experimental procedures for detailed information about the siRNA library). To visualize the effect of gene knockdown on mitotic progression, we fixed the cells 48 h after siRNA transfection and determined the mitotic index by staining the cells using the mitotic marker phospho-Histone H3 (Fig. 1a, b). The screen was performed in both parental HeLa cells and HeLa-derived EICs to identify genes that specifically arrest EICs in mitosis. EICs were always cultured in the presence of the Eg5-inhibitor S-trityl-L-cysteine (STLC) (Debonis et al. 2004). As positive controls in our screen setup, we used siRNA targeting Eg5 to specifically arrest the parental cells in mitosis and siRNAs targeting Kif15 to specifically arrest the EICs in mitosis (Fig. 1b). We used siRNAs targeting the Hec1 gene, encoding an essential outer-kinetochore (KT) component, as a second positive control, since its depletion leads to a mitotic arrest in both cell lines (Fig. 1b). GAPDH siRNA was used as a negative control for both cell lines (Fig. 1b). The primary screen was performed in duplicate in both cell lines, and the results from both screens were analyzed using CellHTS2 and normalized using sample-based normalization (Fig. 1c, Boutros et al. 2006), see experimental procedures for more detailed information about the analysis method). Functional gene-association network analysis using STRING software indicated that the top hits in both cell lines included many genes known to be involved in essential mitotic processes (Szklarczyk et al. 2011). These hits included genes encoding essential KT components, microtubule-associated proteins (MAPs), and centrosomal components (Supplementary Fig. S1 and S2). Unexpectedly, while Kif15 was one of the strongest hits in our screen, we did not identify BicD2 and CENPF in our screen, for which we previously showed that they specifically arrested EICs in mitosis (Raaijmakers et al. 2012). This could be caused by the fact that we previously incubated BicD2 and CENPF siRNAs for 72 h to observe an increased effect on monopolar spindle formation, while in the present setup, 48 h of incubation time was used. In order to identify genes specifically arresting one of the two cell lines, we subtracted the normalized mitotic index of the parental cells from the normalized mitotic index of the EICs (Fig. 1d and Table S1). The scores after subtraction of the normalized mitotic index were plotted and showed Eg5 and Kif15 as clear outliers, specific for the parental cells and the EICs, respectively (Fig. 1d and Table S1). We selected a total of 248 EIC-specific genes for follow-up screening (Table S1). After subtraction of the mitotic indexes, the selected genes had a normalized difference of at least 8 for the EIC-specific genes. The hits from the primary screen were rescreened in triplicate using a similar setup as the primary screen (Fig. 1e). Consistently, we identified Kif15 to be one of the strongest hits after analysis (Fig. 1e and Table S2). We selected 81 genes from the secondary screen with a normalized difference of at least 10 (Table S2). We further validated the EIC-specific hits by performing siRNA pool deconvolution (Fig. 1e, and experimental procedures). From these 85 candidates, seven genes were confirmed on-target using the criteria that at least three siRNAs of the pool scored with a minimum of two times standard deviation (SD) of the siGAPDH (see experimental procedure and Table S2). We tested the seven genes in a final confirmation experiment (Fig. 1f). From these seven initial hits, we were able to confirm three genes, which upon depletion led to a dramatic increase in the mitotic index in the EICs and showed loss of the corresponding protein on western blot (Fig. 1f and Supplementary Fig. S3). The genes identified in the screen, Aurora A, Kif2C (MCAK), and Kif18b (Fig. 1g) where all previously implicated in mitosis, but their exact contribution to bipolar spindle assembly is unclear.

### MCAK, Kif18b, and Aurora A are essential for bipolar spindle assembly in EICs and in cells with reduced Eg5-activity

**Fig. 2:**
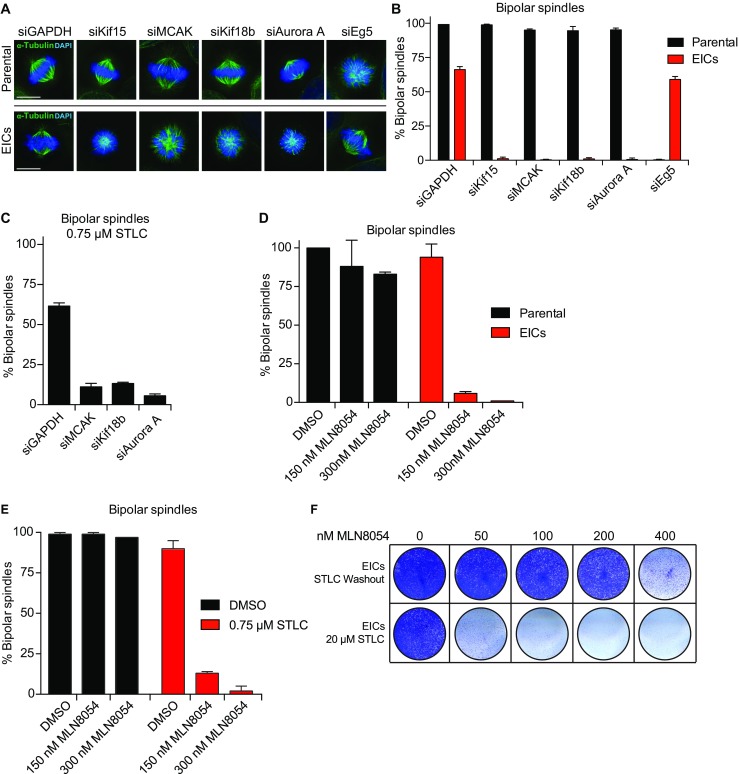
MCAK, Kif18b, and Aurora A are essential for bipolar spindle assembly in EICs and cells with reduced Eg5-activity. **a** Parental and EICs HeLa cells were transfected with the indicated siRNAs, fixed and stained for α-tubulin. DAPI was used to stain the DNA. **b** Percentage of bipolar spindles from the cells treated in (**a**) (*n* > 100 per condition). **c** Parental cells were transfected with the indicated siRNAs and treated for 5 h with 0.75 μM STLC. The cells were fixed and stained as in (**a**) and the percentage of bipolar spindles was scored (*n* > 100 per condition). **d, e** Parental and EICs HeLa cells were treated with the indicated concentrations of MLN8054 and fixed as in (**a**). The percentage of bipolar spindles was scored (*n* > 100 per condition). **f** Colony formation assay of EICs. The cells were treated for 5 days with the indicated drug combination, fixed with methanol and stained with crystal violet. STLC was washed out at the start of the experiment. Results in (**b, c, d, e**) are averages of at least three independent experiments. *Error bars* represent s.d. *Scale bars* in (**a**) represent 10 μm

In order to characterize the cause of the increased mitotic index upon depletion of the different hits in the EIC’s, we depleted MCAK, Kif18b, and Aurora A from parental and EICs and scored the percentage of bipolar spindles (Fig. 2a, b). Similar to Kif15 depletion, the EIC-specific hits from the screen efficiently blocked bipolar spindle assembly while their depletion did not affect spindle bipolarity in the parental cells, explaining the EICs-specific mitotic index increase in the screen (Fig. 2a, b). Next, we determined if the contribution of MCAK, Kif18b, and Aurora A to bipolar spindle assembly was restricted to EICs or if they also contribute to bipolar spindle assembly in parental cells. To test this, we partially inhibited Eg5 activity in parental HeLa cells using a low dose (0.75 μM, (Raaijmakers et al. 2012) of STLC. Similar to our results in EICs, siRNA depletion of MCAK, Kif18b, and Aurora A in parental HeLa cells, treated with a low dose of STLC, fully blocked bipolar spindle assembly (Fig. 2c). This indicates that the function of these proteins in bipolar spindle assembly is not restricted to EICs, but that their function is masked by the major centrosome-separating force produced by Eg5 in normal cells.

Both Eg5 and Aurora A inhibitors are promising anti-cancer drugs that are currently investigated in a number of clinical trials (Lens et al. 2010; Janssen and Medema 2011). The dramatic increase in monopolar spindles in cells treated with Aurora A siRNA and Eg5-inhibitors made us wonder if combined treatment of Eg5 inhibitors with Aurora A inhibitors might lead to a synergistic effect in blocking bipolar spindle formation and as a long-term consequence, in decreased cell proliferation. To test this, we treated both parental and EICs with the selective Aurora A inhibitor MLN8054 (Manfredi et al., 2007). Similar to siRNA treatment, concentrations up to 300 nM MLN8054 did not affect bipolar spindle formation in parental cells, but efficiently blocked bipolar spindle formation in EICs (Fig. 2d) and in parental cells with reduced Eg5 activity (Fig. 2e). In addition, long-term treatment with low doses of MLN8054 (50–100 nM) blocked proliferation in EICs, while similar doses did not affect EICs after removal of STLC (Fig. 2f). Furthermore, long-term treatment of parental HeLa cells with low doses of Eg5 and Aurora A inhibitors that efficiently blocked the formation of bipolar spindles (Fig. 2e) also fully blocked proliferation and induced apoptotic cell dead as shown by the increased levels of cleaved poly(ADP-ribose) polymerase (PARP) by caspase-3 (Supplementary Fig. S4). These data suggest that combining Eg5 and Aurora A inhibitors might have increased efficacy versus monotherapy. In addition, it might prevent the development of resistance to Eg5 inhibitors.

### MCAK, Kif18b, and Aurora A are required for bipolar spindle maintenance in the absence of Eg5 activity

**Fig. 3:**
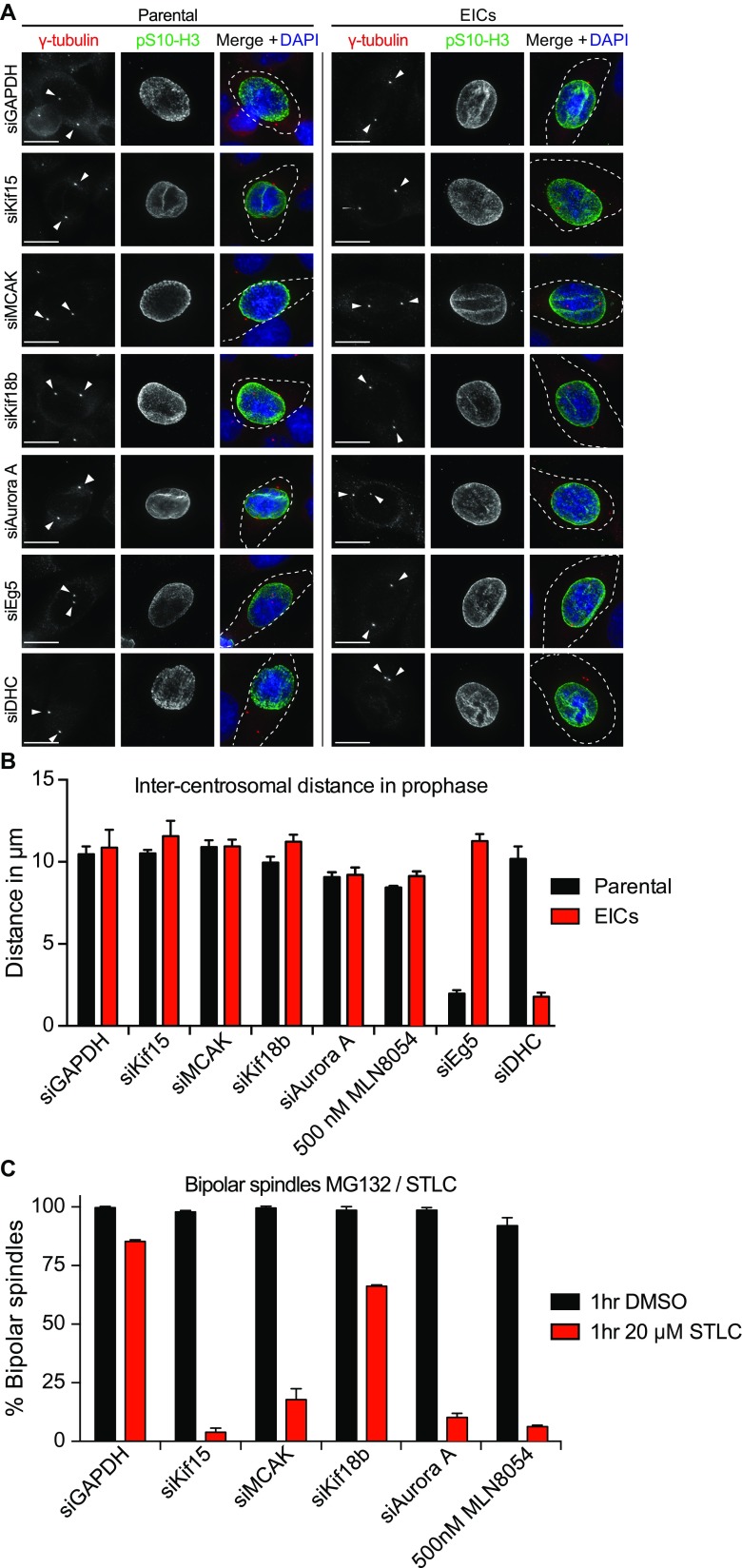
MCAK, Kif18b, and Aurora A are required for bipolar spindle maintenance in the absence of Eg5 activity. **a** Representative images of parental and EICs HeLa cells treated with the indicated siRNAs. Cells were stained for ϒ-tubulin to visualize the centrosomes, phospho-H3 (pH 3) to mark prophase cells and DAPI to visualize the DNA. *Arrowheads* in the pictures mark the centrosomes. **b** Quantification of the inter-centrosomal distance in prophase from the cells in (**a**) (*n* = 45 per condition). **c** Parental HeLa cells were treated with MG132 for 1 h and subsequently treated with 20 μM STLC for an additional hr. Cells were fixed and stained as in (**a**), and the percentage of mitotic cells with a bipolar spindle was scored (*n* > 100 per condition). Quantification of the mitotic timing of HeLa cells expressing H2B-mCherry and GFP-α-tubulin. Cells were treated for 48 h with the indicated siRNAs. Before starting the time-lapse acquisition, cells were arrested in metaphase for 1 h using the proteasome inhibitor MG132. Cells were then treated with 20 μM STLC and images were acquired every 4 min. Results from (**b**) are averages of at least three different experiments. *Error bars* represent s.d. *Scale bars* represent 10 μm

We have previously shown that EICs critically depend on nuclear-envelope (NE) dynein-mediated centrosome separation in prophase and Kif15-activity during prometaphase in order to build a bipolar spindle (Raaijmakers et al. 2012; van Heesbeen et al. 2013). In order to determine in which pathway for bipolar spindle assembly the genes identified in our screen act, we first tested if their depletion affected centrosome separation during prophase. While dynein depletion efficiently blocked prophase centrosome separation in EICs, depletion of MCAK, Kif18b, and Aurora A did not have a significant effect on prophase centrosome separation that could explain the number of monopolar spindles in prometaphase (Fig. 3a, b). This indicates that the function of these proteins in bipolar spindle formation is most likely restricted to prometaphase.

To test if the action of MCAK, Kif18b, and Aurora A is restricted to the assembly of the bipolar spindle during prometaphase, or if their function is also required for the maintenance of the metaphase bipolar spindle, we arrested parental HeLa cells in metaphase using the proteasome inhibitor MG132 and subsequently inhibited all Eg5 activity using a high dose of STLC (Tanenbaum et al. 2009; van Heesbeen et al. 2014). As we have shown previously, control cells maintain a bipolar spindle after treatment with STLC, due to the action of Kif15 (Tanenbaum et al. 2009). Similar to Kif15 depletion, MCAK, Aurora A, and to a lesser extent Kif18b depletion, results in collapse of the bipolar spindle upon Eg5 inhibition (Fig. 3c). Curiously, we find a difference in the number of bipolar spindles that collapsed in the MCAK-depleted cells versus the Kif18b-depleted cells. While MCAK and Kif18b act together to control astral MT dynamics (Tanenbaum et al. 2011a; Stout et al. 2011), previous studies showed that additional, Kif18b-independent roles for MCAK at kinetochores and spindle poles exist (Walczak et al. 2013) and likely contribute to the differences we observed in the number of cells in which we observed collapse of the bipolar spindle. These results indicate that the action of these proteins is not restricted to the assembly of the bipolar spindle, but is also required for maintenance of the metaphase bipolar spindle.

### Excessive astral microtubule nucleation blocks centrosome separation and bipolar spindle assembly

Regulation of MT dynamics during mitosis is a tightly regulated process (Howard and Hyman 2007). The MT motors from the kinesin-13 and kinesin-8 family have both been shown to control MT depolymerization (Walczak et al. 2013). MCAK (kinesin-13) is a non-processive motor that diffuses along the MT lattice to reach the ends of MTs (Helenius et al. 2006) and contains an internal motor domain required for its MT depolymerizing activity (Hunter et al. 2003). Besides that, MCAK can also track the growing plus-ends of MTs through a direct interaction with EB1 (Moore et al. 2005; Lee et al. 2008). Previous studies implicated a role for kinesin-13 family members in mitosis, including the regulation of spindle bipolarity (Ganem and Compton 2004; Kollu et al. 2009) and positioning of the mitotic spindle (Rankin and Wordeman 2010). However, the mechanisms by which MCAK contributes to Eg5-independent bipolar spindle assembly are still unclear. In contrast to kinesin-13, kinesin-8 motors contain a N-terminal motor domain and have shown to be processive, plus-end-directed motors (Gupta et al. 2006; Varga et al. 2006; Mayr et al. 2007), that depolymerize MTs at the plus-ends in a length-dependent manner (Varga et al. 2006; Varga et al. 2009). Kif18b has also been shown to accumulate at MT plus-ends through a direct interaction with EB1 (Tanenbaum et al. 2011a; Stout et al. 2011). In addition, Kif18b interacts with MCAK, hereby promoting the plus-end accumulation of each other (Tanenbaum et al. 2011a). While MCAK regulates MT depolymerization at different locations in the cell, including kinetochores, centrosomes, and astral MTs (Andrews et al. 2004; Kline-Smith et al. 2004; Tanenbaum et al. 2011b), the localization of Kif18b is negatively regulated by Aurora kinases and has only been found at the plus-tips of astral MTs (Tanenbaum et al. 2011a). Taking into account that Kif18b localization is restricted to astral MTs and MCAK is a non-processive motor, it is unlikely that they act in sliding anti-parallel MTs. However, considering that MCAK and Kif18b both regulate astral MT depolymerization by forming a mitosis-specific complex (Tanenbaum et al. 2011a), we wondered if astral MT length control could influence bipolar spindle assembly.

**Fig. 4:**
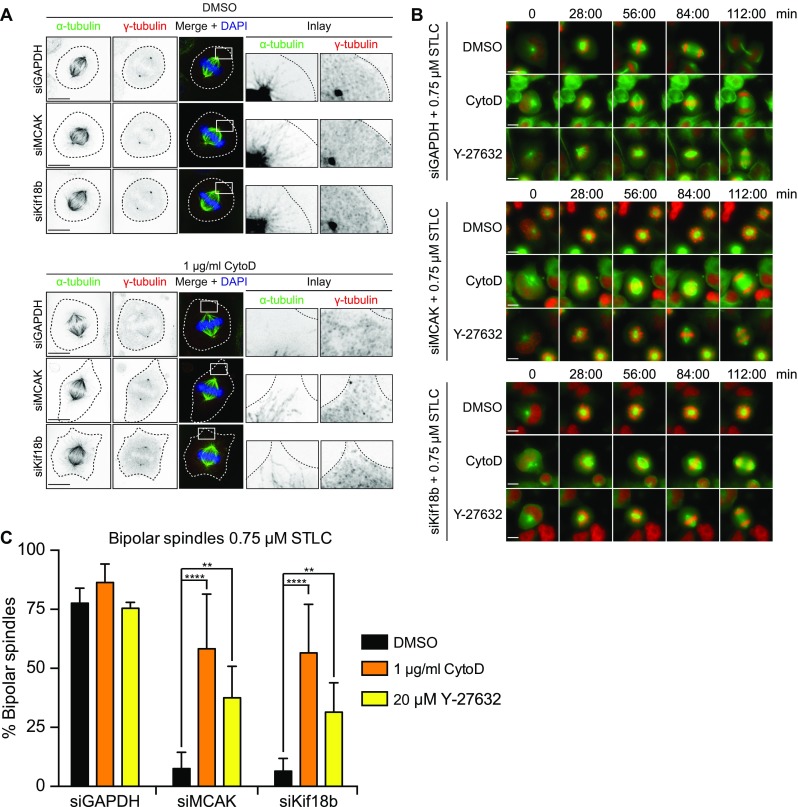
Excessive astral MT nucleation block centrosome separation and bipolar spindle formation. **a** Representative images of parental HeLa cells treated with the indicated siRNAs and drug combinations. Forty-eight hours after siRNA transfection, the cells were fixed and stained for α-tubulin and ϒ-tubulin. DAPI was used to stain the DNA. The *dotted line* indicates the cell cortex. *Boxed area* is enlarged in the inlay. Note the deformation of the cortex by astral MTs in the cytochalasin D-treated cells. **b** Representative stills of parental HeLa cells, expressing H2B-mCherry and GFP-α-tubulin, treated with the indicated siRNAs and drugs combinations. Note the rescue in spindle bipolarity in MCAK and Kif18b-depleted cells after treatment with cytochalasin D and Y-27,632. **c, d** Quantification of the percentage of bipolar spindles from the cells in (**b**). *n* > 100 per condition. **P < 0.01 and ****P < 0.0001. *p* values calculated using two-way ANOVA. Results in C are averages of at least three different experiments. *Error bars* represent s.d. *Scale bars* represent 10 μm

Depletion of either MCAK or Kif18b results in the formation of excessive and long astral MTs (Fig. 4a and (Kline-Smith et al. 2004; Rankin and Wordeman 2010; Tanenbaum et al. 2011a; Stout et al. 2011). Furthermore, a single astral MT can grow towards the cell cortex where it can push against the cell membrane for a short amount of time before it undergoes catastrophe and subsequent shrinkage (Tran et al. 2001). During this short contact time, a single MT can exert a substantial amount of force, that is comparable to the force exerted by a single kinesin molecule (Visscher et al. 1999; Janson et al. 2003). Indeed, upon depletion of MCAK and Kif18b, we observed high numbers of astral MTs reaching the cortex and buckling of astral MTs indicating continuous polymerization and force generation by these astral MTs (Fig. 4a). To test if the excessive astral MTs that form in the absence of MCAK and Kif18b might generate forces by continuous growth against the cortex, we lowered cortical membrane tension by disrupting the actomyosin cytoskeleton using cytochalasin D. This prevents polymerizing astral MTs from generating forces via the cortical membrane on spindle poles, and polymerizing astral MTs would rather deform the cortex upon loss of the actomyosin network. Upon disruption of the actomyosin network in MCAK or Kif18b depleted cells, we indeed observed cortical membrane protrusions in which astral MTs continued to polymerize (Fig. 4a, middle panel, Supplementary Fig. S5 and movie S1–S4). Disruption of the actomyosin network hardly affected the number of bipolar spindles formed in control-treated cells in which Eg5-activity was partially inhibited (Fig. 4b, c). However, disruption of the actomyosin network in MCAK- or Kif18b-depleted cells produced an increase in the amount of bipolar spindles (Fig. 4b, c), indicating that at least some of the forces that perturb bipolar spindle formation in MCAK- or Kif18b-depleted cells depend on cortical tension. In addition to cytochalasin D treatment, we used a second strategy to lower cortical tension by inhibiting Rho-kinase using the small molecule Y-27632 (Tinevez et al. 2009). Similar to cytochalasin D treatment, we observed a pronounced rescue in the number of bipolar spindles after MCAK and Kif18b depletion when we combined partial Eg5 inhibition with Rho-kinase inhibition (Fig. 4b, c). Furthermore, while cytochalasin D treatment was extremely toxic in EICs and prevented entry into mitosis, we could also partially rescue bipolar spindle assembly in EICs after depletion of either MCAK or Kif18b combined with Rho-kinase inhibition (Supplemental Fig. S6). Although we only observed a minor increase in the number of bipolar spindles after disruption of the cortical actomyosin network and, due to technical constrains, we were not able to convincingly image MT-cortical interaction, our data suggest that upon disruption of the cortical actomyosin network, astral MTs cannot generate sufficient force on the centrosomes to counteract the forces that drive the separation of centrosomes.

### Phosphorylation of S1169 by Aurora A is required to target Kif15 to the spindle

The role of Aurora A in centrosome separation and bipolar spindle formation is controversial. Early studies in Drosophila showed that mutations in Aurora A led to centrosome separation defects and monopolar spindle formation (Glover et al. 1995). Consistently, studies in mouse embryonic fibroblasts showed that Aurora A deletion led to the formation of monopolar spindles (Cowley et al. 2009). In contrast, Aurora A deletion in chicken DT40 cells led to the formation of small bipolar spindles (Hégarat et al. 2011) and studies in human cells observed a wide variety of phenotypes, including chromosome misalignments, multipolar spindles, and monopolar spindles (Marumoto et al. 2003; Hoar et al. 2007). The discrepancies observed between model systems could be explained by the methods used to inactivate or deplete Aurora A from cells or by different contributions of parallel pathways involved in centrosome separation and bipolar spindle assembly (Smith et al. 2011). Despite the fact that a wide variety of Aurora A substrates have been identified (Lens et al. 2010; Hochegger et al. 2013), clear downstream targets involved in centrosome separation and bipolar spindle assembly are poorly understood. Eg5 was described to be phosphorylated by Aurora A in Xenopus (Giet et al. 1999), but since we identified Aurora A in an Eg5-independent background, this cannot be its only target for its function in bipolar spindle assembly. Furthermore, centrosome maturation and MT nucleation might also indirectly affect centrosome separation, although these function are likely not affected in our system, since we did not observe major defects in prophase centrosome separation in both normal cells and EICs (Fig. 3a, b). At last, the fact that we observed a rapid bipolar spindle collapse in cells in which we blocked both Eg5 and Aurora A activity simultaneously (Fig. 3c) points towards a target that is also involved in the maintenance of the bipolar spindle.

**Fig. 5:**
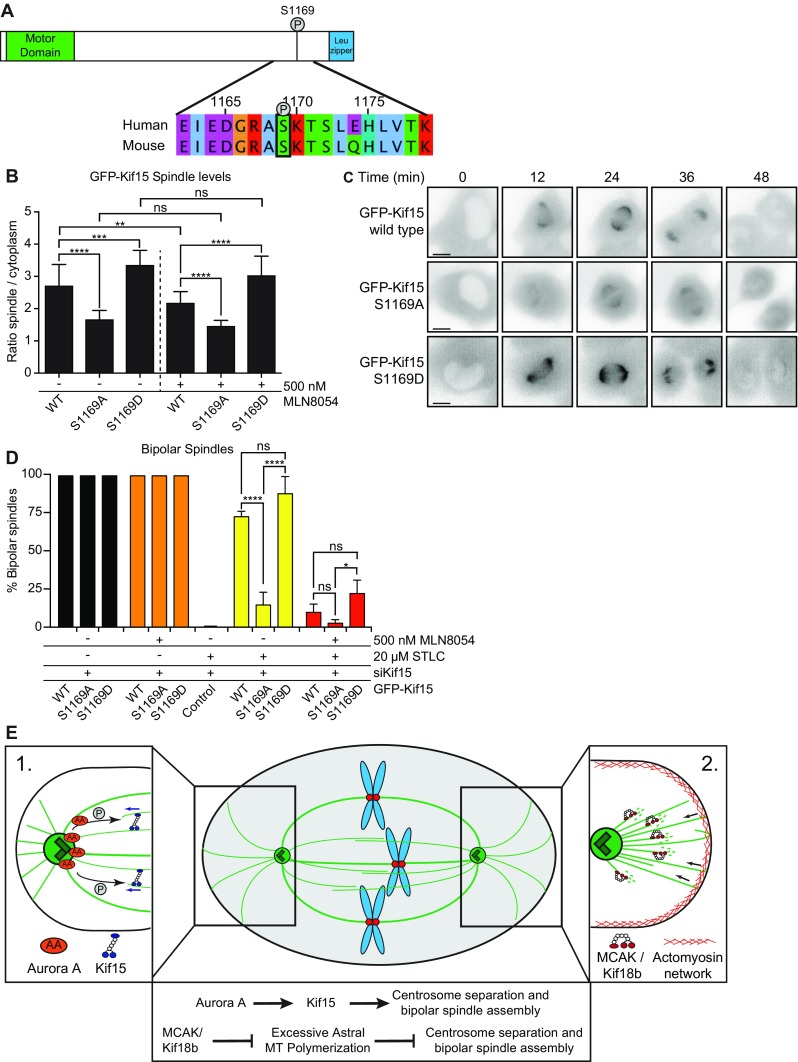
Phosphorylation of S1169 by Aurora A is required for targeting Kif15 to the spindle. **a** Schematic representation of Kif15. The conserved domain spanning serine 1169 is shown in the inlay. **b** Spindle levels of GFP-Kif15 phospho-mutants. U2OS cells were transfected with the indicated construct and treated as indicated. The spindle level of the different GFP-Kif15 mutants was determined by dividing the spindle levels over the levels of the cytoplasm (*n* = 20 cells) **P < 0.01; ***P < 0.001; ****P < 0.0001; *ns* not significant. *p* values calculated with one-way ANOVA. **c** Representative stills of U2OS cells expressing the annotated GFP-Kif15 construct. Note the reduced spindle levels after expression of the GFP-Kif15 S1169A mutant. **d** Quantification of the percentage of bipolar spindles in U2OS cells. The cells were transfected with the annotated GFP-Kif15 constructs and treated as indicated. Results in (**d**) are averages of at least three different experiments (*n* = 90 per condition) *P < 0.1; ****P < 0.0001; *ns* not significant. *p* values calculated with two-way ANOVA. *Error bars* represent s.d. *Scale bar* represents 10 μm. **e** Model about the contribution of MCAK, Kif18b and Aurora A in bipolar spindle assembly. **1** Aurora A phosphorylates Kif15 to target it to the spindle, hereby promoting its function in bipolar spindle assembly. **2** MCAK and Kif18b control the number and length of astral MTs, hereby preventing astral MTs from generating forces at the cortex that counteract centrosome separation and bipolar spindle assembly

Since Kif15 and Aurora A depletion have overlapping phenotypes in EICs, we wondered if Aurora A might directly regulate Kif15. To identify potential Aurora A phosphorylation sites in Kif15, we performed an in vitro kinase assay using recombinant Kif15 and Aurora A. Mass-spectrometry of Kif15 identified multiple residues being phosphorylated by Aurora A (Supplementary Fig. S7). One particular conserved residue we identified, serine 1169, matched the Aurora A phosphorylation consensus sequence R-X-[S/T] (Fig. 5a and Supplementary Fig. S7; (Kettenbach et al. 2011)) and was also previously identified in vivo to be specifically phosphorylated during mitosis (Olsen et al. 2010). To examine the role of S1169 phosphorylation on Kif15 by Aurora A, we expressed GFP-tagged versions of mouse Kif15 (Tanenbaum et al. 2009), as well as a non-phosphorylatable Kif15-S1169A and phosphomimetic Kif15-S1169D mutant in U2OS cells. All constructs were expressed at similar levels in U2OS cells (Supplementary Fig. S8), and we selected cells expressing equal amounts of GFP-tagged Kif15 to determine the spindle localization. While the wild-type and Kif15-S1169D localized normally to the spindle (Fig. 5b and movie S5 and S7), recruitment of the S1169A mutant to the spindle was markedly reduced (Fig. 5b and movie S6). This suggests that S1169 phosphorylation on Kif15 by Aurora A promotes its spindle localization during mitosis.

Next, we tested if overexpression of the Kif15 mutants could bypass the requirement of Eg5 in cells where we depleted endogenous Kif15 (Tanenbaum et al. 2009). As expected, overexpression of the different constructs did not affect bipolar spindle assembly in the absence or presence of MLN8054 (Fig. 5c, black and orange bars). However, while full Eg5 inhibition efficiently blocked bipolar spindle formation in control-transfected cells (Fig. 5c, middle bar), overexpression of wild-type and S1169D-mutated Kif15 fully restored bipolar spindle formation in Eg5-inhibited cells (Fig. 5c, yellow bars). In contrast, expression of S1169A-mutated Kif15 did not restore bipolar spindle formation, indicating that this mutant is not capable to bypass the Eg5 requirement (Fig. 5c, yellow bars). Finally, when we combined Eg5 inhibition with partial Aurora A inhibition (500 nM MLN8054), we observed a pronounced decrease in the amount of bipolar spindles when we overexpressed wild-type Kif15 (Fig. 5c, red bars). Strikingly, the S1169D mutant was still partially active and about 25 % of the cells formed bipolar spindles upon combined inhibition of Eg5 and Aurora A (Fig. 5c, red bars). These results indicate that Aurora A directly regulates Kif15 by targeting it to the spindle during mitosis through phosphorylation on S1169.

## Discussion

Here, we performed a genome-wide siRNA screen in parental and EICs cells to identify novel factors involved in Eg5-independent bipolar spindle formation. Using our setup, we identified three genes required for bipolar spindle assembly in EICs. We show that the microtubule motors MCAK and Kif18b are required for bipolar spindle assembly in EICs and normal cells with reduced Eg5-activity. In contrast to Eg5, which directly drives bipolar spindle assembly by sliding antiparallel MTs apart (Kashina et al. 1996; Kapitein et al. 2005; Tanenbaum et al. 2009), we show evidence that the contribution of MCAK and Kif18b to bipolar spindle assembly is likely mediated by their function in regulating the length and number of astral MTs during mitosis. Although we cannot rule out additional contributions of MCAK and Kif18b in bipolar spindle assembly, we hypothesize that in the absence of either MCAK or Kif18b, the tight balance in astral MTs nucleation and depolymerization is lost and excessive astral MTs generate inward pushing forces on centrosomes when these MTs collide with the cortex (Fig. 5e). Under normal conditions, these forces are not sufficient to counteract outward forces, but when Eg5 activity is compromised, growing astral MTs generate forces on the cortex to counteract the remaining centrosome separation forces by Eg5 and Kif15. Our results indicate that MCAK and Kif18b have an equal and non-redundant contribution in regulating astral MT dynamics. However, we did observe a more rapid collapse of the preassembled bipolar spindle after MCAK depletion compared to Kif18b, suggesting possible Kif18b-independent involvement of MCAK. Furthermore, interfering with cortical tension could rescue bipolar spindle assembly for both MCAK and Kif18b only to a limited extend. We therefore cannot exclude that additional functions of MCAK and Kif18b contribute to bipolar spindle assembly. In line with that, MCAK was previously shown to be involved in the regulation of KT-MT turnover, and this function might also contribute to bipolar spindle assembly and maintenance (Andrews et al. 2004; Kline-Smith et al. 2004). In addition to MCAK and Kif18b, we identified Aurora A in our screen to be required for bipolar spindle assembly in EICs. Although Aurora A was previously identified to act synergistically lethal with Eg5 inhibitors, its downstream targets for controlling centrosome separation are poorly understood (Ma et al. 2014). We now show that Aurora A functions in bipolar spindle formation by controlling the localization and activity of Kif15. Spindle localization of Kif15 is decreased upon inhibition of Aurora A. While Kif15 function is under normal conditions redundant for bipolar spindle assembly, its function is essential for EICs and cells with reduced Eg5-activity (Tanenbaum et al. 2009; Raaijmakers et al. 2012; van Heesbeen et al. 2014), explaining the high sensitivity for Aurora A inhibition under this condition. Due to conflicting results from recent studies (Sturgill and Ohi 2013; Drechsler et al. 2014; Sturgill et al. 2014), it is currently unclear how Kif15 functions at the molecular level and whether Kif15 acts on antiparallel MTs in the spindle. Interestingly, both Aurora A and Kif15 require TPX2 for their function and depletion of TPX2 prevents spindle targeting of Kif15. How phosphorylation of S1169 contributes to spindle targeting of Kif15 is still unclear, but it might affect the interaction of TPX2 with the C-terminal leucine zipper (Tanenbaum et al. 2009), or affect the previously proposed non-motor MT-binding domain of Kif15 (Sturgill et al. 2014).

Although we find direct phosphorylation of Kif15 by Aurora A, it has likely more targets required for bipolar spindle assembly. We observed that cells expressing high levels of S1169A Kif15 were still able to form bipolar spindles. This could indicate that additional Aurora A phosphorylation sites on Kif15 are present. Aurora A also contributes to MT nucleation and KT-MT stability (Kinoshita et al. 2005; Ertych et al. 2014), which has been shown to contribute to bipolar spindle assembly and maintenance (Sturgill and Ohi 2013; van Heesbeen et al. 2014). Most likely, a combination of regulating MT dynamics and kinesins like Kif15, explains the synergistic effect we see after combined inhibition of Eg5 and Aurora A.

Both Eg5 and Aurora A inhibitors are currently being tested as potential anti-cancer drugs in clinical trials (Rath and Kozielski 2012; Malumbres and Pérez de Castro 2014). In order to enhance efficacy, we propose that combination therapy of Eg5 and Aurora A inhibitors might be beneficial because of three main reasons. First, the combined treatment shows a very strong synergistic effect in the formation of monopolar spindles, even when both proteins are only partially inhibited. Second, the development of resistance mechanisms for Eg5 inhibitors (Tanenbaum et al. 2009; Raaijmakers et al. 2012) will likely be prevented by combining the Eg5 and Aurora A inhibitors. And last, there are currently no Kif15 inhibitors available, which makes Aurora A inhibitors currently the most attractive candidate to increase the efficacy for Eg5 inhibitors. Taken together, we unraveled new mechanisms for bipolar spindle assembly that might have promising translational applications.

## Experimental procedures

### Screen setup, analysis, and normalization

The human ON-TARGETplus siRNA SMARTpool library (Dharmacon) was used for the primary screen. The siRNAs for the secondary screen were manually picked and re-tested. For the deconvolution screen, the four single siRNAs of the SMARTpool were tested separately. The primary screen was performed in duplicate, the secondary and deconvolution screen were screened in triplicate.

For the primary and secondary screen, siRNA libraries were aliquoted in a 384-well format using a Sciclone liquid handling robot (Caliper). Deconvolution screen was performed in a 96-well format. A final concentration of 20 nM siRNA per well was used. Per transfection, 0.075 μl RNAiMAX (Invitrogen) and 10 μl Opti-MEM (GIBCO) were added to the siRNA and incubated for about 20 min. One thousand five hundred cells diluted in 40 μl media were added to the wells after incubation of the transfection reagents, using a MultiDrop Combi bulk dispenser (Thermo).

After 48 h of culturing, the cells were fixed for 10 min using a final concentration of 4 % formaldehyde (3× formaldehyde in PBS was added to the wells). Fixation reagent was added using a Multidrop Combi bulk dispenser (Thermo), primary and secondary antibodies were added using the Sciclone (Caliper), and all washing step was performed in an AquaMax 2000 plate washer (MDC).

After staining of the wells, the mitotic index of the wells was analyzed using a Cellomics Arrayscan VTI (Thermo Scientific) using a 10× (0.50 NA) objective. Four images were acquired per well. Image analysis was performed using Cellomics Target Activation Bioapplication (Thermo Scientific). Cells were identified based on the DAPI staining and were scored to be mitotic if the phospho-Histone H3 signal reached a set threshold.

The raw mitotic index data was normalized using the CellHTS2 package (Boutros et al. 2006). For the primary screen, sample-based normalization was used. For the secondary and deconvolution screen, control-based normalization was used. After subtraction of the normalized mitotic indexes, the top genes (EICs specific) from the primary screen, that had a normalized difference in the mitotic index of 8, were selected for the secondary screen. Similar criteria were used for the secondary screen. For the deconvolution screen, a siRNA duplex was confirmed on-target when the increase in the normalized mitotic index was >2 times standard deviation of the negative control (siGAPDH) in all replicates.

### Cell culture, transfection, and drug treatment

Cells were cultured in DMEM (GIBCO), supplemented with 6 % fetal calf serum, 100 U/ml penicillin and 100 μg/ml streptomycin. siRNAs were transfected using RNAiMax (Invitrogen) according to the manufactures guidelines. DNA was transfected using FuGENE 6 (Promega) according to the manufactures guidelines. The following siRNAs were used in this study: GAPDH OTP SMARTpool (Dharmacon), MCAK/Kif2C OTP SMARTpool (Dharmacon), Kif18b OTP SMARTpool (Dharmacon), Aurora A OTP SMARTpool (Dharmacon), Eg5/Kif11 OTP SMARTpool (Dharmacon), Kif15/HKlp2 OTP SMARTpool (Dharmacon) and custom siRNA (GAATGACTGATGAAGTCGA, Ambion, Tanenbaum et al. 2009), and Dynein heavy chain (Walczak et al. 1996). The following expression constructs were used in this study: mouse pTON-bEGFP-Kif15 (Tanenbaum et al. 2009). Phosphomutants of Kif15 were generated using site-directed mutagenesis. STLC (Sigma) was used at a concentration of 20 and 0.75 μM for EICs and parental cells, respectively. MLN8054 (Millenium Pharmaceuticals), MG132 (Sigma), nocodazole (Sigma), cytochalasin D (Sigma), and Y-27632 (Sigma) were all used at the indicated concentrations.

### Immunofluorescence

Cells were grown on 10-mm glass coverslips and pre-extracted for 60 s in PEM buffer (100 mM PIPES, 10 mM EGTA, 1 mM MgCl, and 0.1 % Triton X-100) followed by fixation in 4 % formaldehyde in PEM buffer with 0.3 % Triton X-100 for 10 min at room temperature. The following primary antibodies were used: α-tubulin antibody (Sigma) was used 1:10,000, phospho-H3 (Serine 10, Millipore) was used at 1:1500, γ-tubulin antibody (Abcam) was used 1:500. All antibodies were incubated overnight at 4 °C. Secondary antibodies (Alexa 488, 568, 647, Molecular Probes) were incubated for 1 h at room temperature. DAPI was added before mounting using ProLong Gold (Invitrogen). Images were acquired using a Deltavision deconvolution microscope (Applied Precision) with a 60× (NA 1.42) or a 100× (NA 1.40) oil objective, Softworx (Applied Precision), Fiji image software and Adobe Photoshop and Illustrator CS6.

### Time-lapse microscopy

Cells were plated on 8-well glass-bottom dished (LabTek). Cells were imaged using a Delatavision deconvolution microscope (Applied Precision) equipped with a heated chamber and cultured in L-15 CO_2_-independent medium (GIBCO). Images were acquired every 4 min using a 20× (NA 0.25) objective. Z-stacks were acquired with 2.5-μm intervals. Images were processed using Softworx (Applied Precision), Fiji image software and Adobe Photoshop and Illustrator CS6.

### Colony formation

Cells were plated at a density at a density of 10,000 cells per well in a 48-well plate, treated as indicated and grown for about 7 days. Cells were fixed and stained using methanol and crystal violet.

### Western blot

Cells were counted and lysed using Laemmli buffer (120 mM Tris pH 6.8, 4 % SDS, 20 % glycerol). Protein levels were analyzed by western blot. The following antibodies were used: MCAK (Walczak et al. 1996) was used 1:1000, Kif18b (Tanenbaum et al. 2011a) was used 1:500, Aurora A (Cell Signaling) was used 1:1000, α-tubulin (Sigma) was used 1:10,000, Cdk4 (Santa Cruz) was used 1:2000, and cleaved PARP (Cell Signaling) was used 1:1000, Hsp90 (Santa Cruz) was used 1:2000.

### Identification of phosphorylation sites

Five micrograms of recombinant mouse His-GFP-Kif15, purified from SF9 cells was incubated with 0.75 μg recombinant human His-Aurora A (Enzo Lifesciences) for 30 min in kinase buffer (50 mM Tris pH 7.5, 15 mM MgCl, 2 mM EGTA, 0.5 mM Vanadate, 1 mM DTT) in the presence of 60 μM ATP. Kinase assay using recombinant His-Aurora A and recombinant human histone-3 (NEB) served as a control. Phosphorylation sites on Kif15 were identified by mass-spectrometry with a nano-LC-LTQ-Orbitrap (Thermo Scientific).

## Electronic supplementary material

Table S1 (XLSX 2724 kb)

Table S2 (XLSX 38 kb)

Figure S1 (PDF 13945 kb)

Figure S2 (PDF 11714 kb)

Figure S3 (PDF 309 kb)

Figure S4 (PDF 1401 kb)

Figure S5 (PDF 232 kb)

Figure S6 (PDF 382 kb)

Figure S7 (PDF 1115 kb)

Figure S8 (PDF 166 kb)

Movie S1 (AVI 543 kb)

Movie S2 (AVI 574 kb)

Movie S3 (AVI 609 kb)

Movie S4 (AVI 528 kb)

Movie S5 (AVI 114 kb)

Movie S6 (AVI 119 kb)

Movie S7 (AVI 120 kb)
