# Supplementary material for: Aurora A, MCAK, and Kif18b promote Eg5-independent spindle formation
Source: Chromosoma. 2016 Jun 29;126(4):473–86. doi: 10.1007/s00412-016-0607-4 (PMC5509784; doi:10.1007/s00412-016-0607-4)
Supplement: Supplementary file 3 — (PDF 13945 kb) [file 412_2016_607_MOESM3_ESM.pdf]

Supplementary Figure 1. Functional interaction network of the 200 top hits in parental cells.

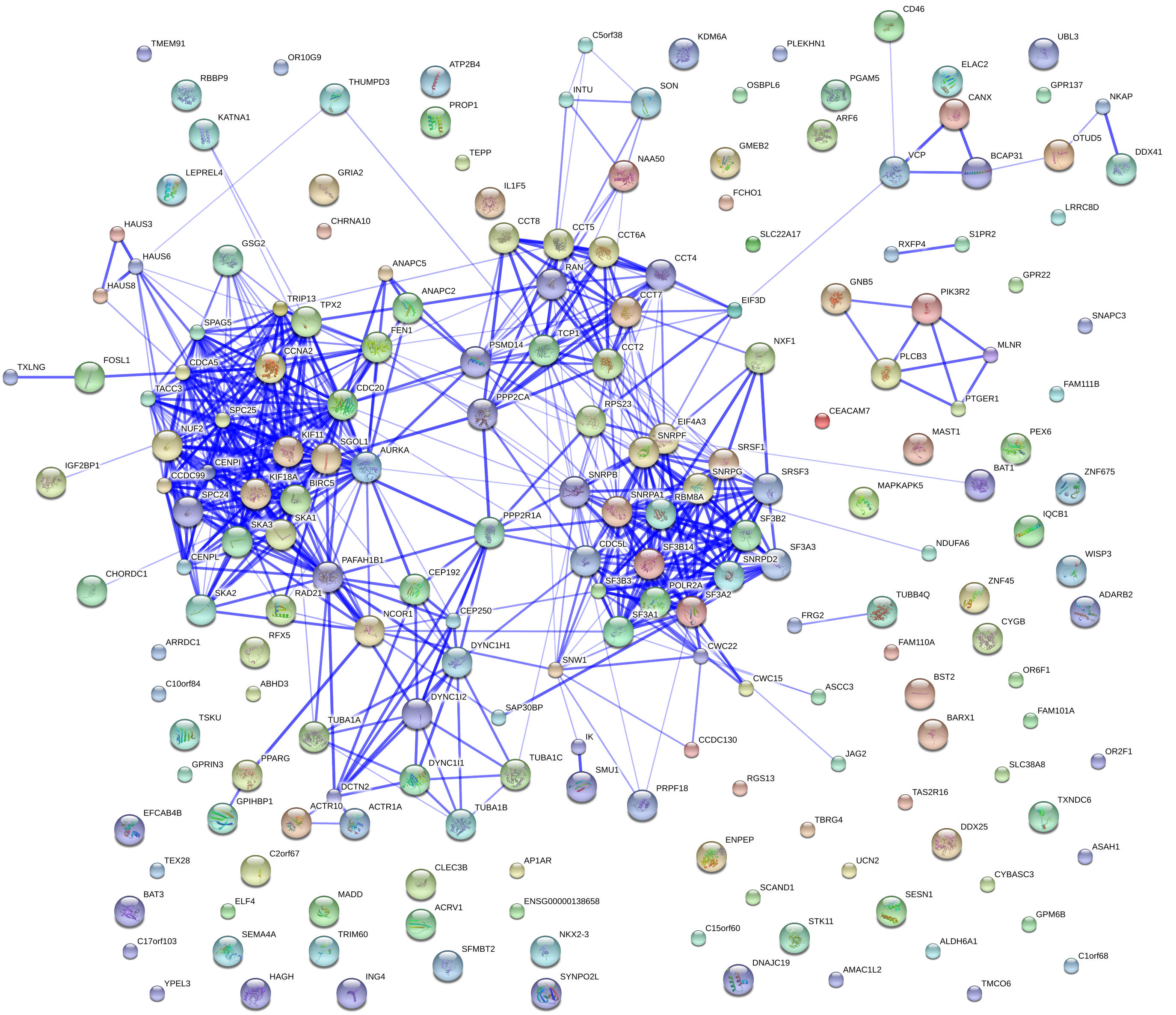

Supplementary Figure 1. Functional interaction network of the 200 top hits in parental cells.
Top hits and interaction of the genes scored in parental cells, retrieved by STRING association.
