# Supplementary material for: Aurora A, MCAK, and Kif18b promote Eg5-independent spindle formation
Source: Chromosoma. 2016 Jun 29;126(4):473–86. doi: 10.1007/s00412-016-0607-4 (PMC5509784; doi:10.1007/s00412-016-0607-4)
Supplement: Supplementary file 4 — (PDF 11714 kb) [file 412_2016_607_MOESM4_ESM.pdf]

Supplementary Figure 2. Functional interaction network of the 200 top hits in EICs.

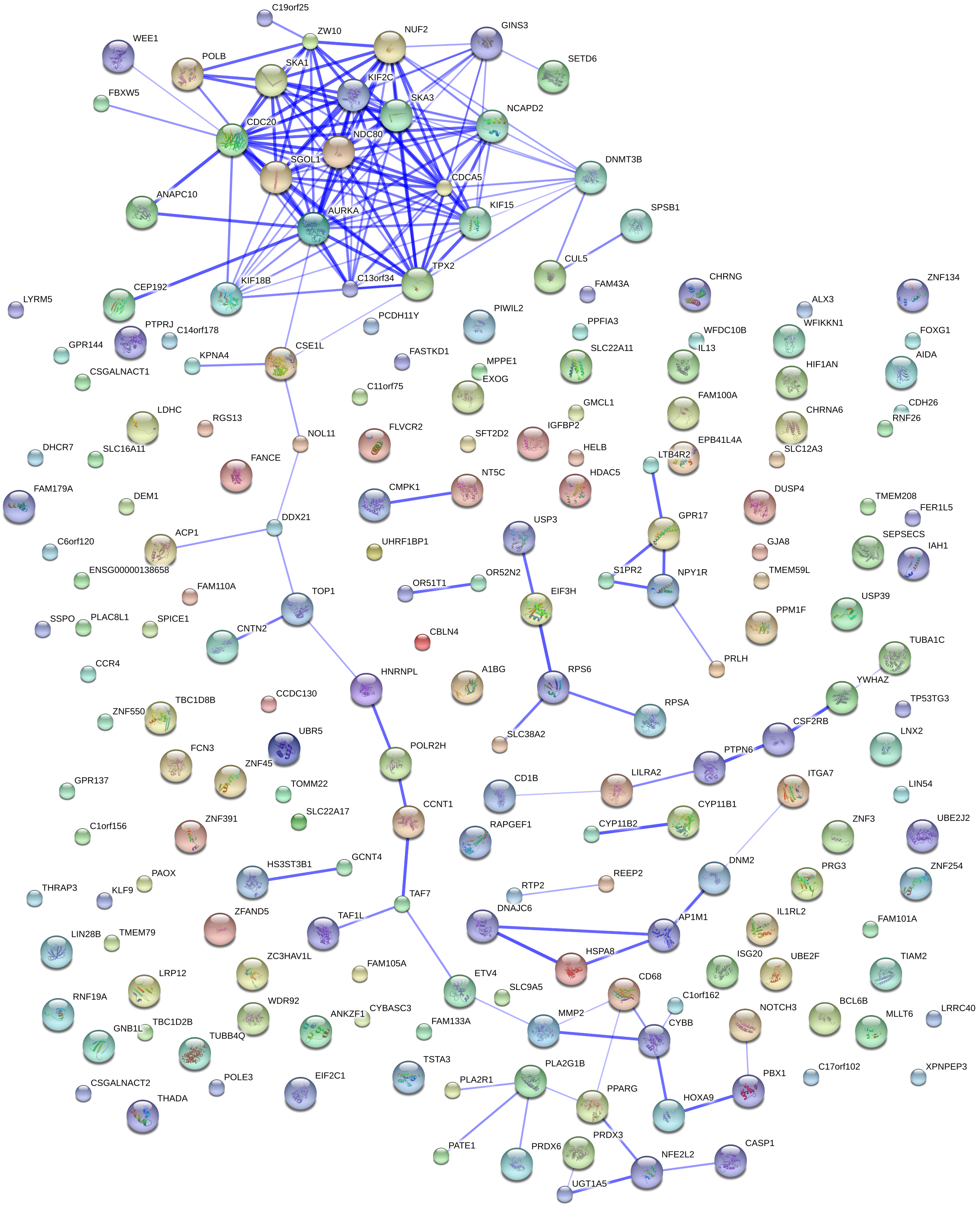

Supplementary Figure 2. Functional interaction network of the 200 top hits in EICs.
Top hits and interaction of the genes scored in EICs, retrieved by STRING association.
