# Supplementary material for: Aurora A, MCAK, and Kif18b promote Eg5-independent spindle formation
Source: Chromosoma. 2016 Jun 29;126(4):473–86. doi: 10.1007/s00412-016-0607-4 (PMC5509784; doi:10.1007/s00412-016-0607-4)
Supplement: Supplementary file 5 — (PDF 309 kb) [file 412_2016_607_MOESM5_ESM.pdf]

Supplementary Figure 3. Knockdown confirmation of the siRNAs used in this study.

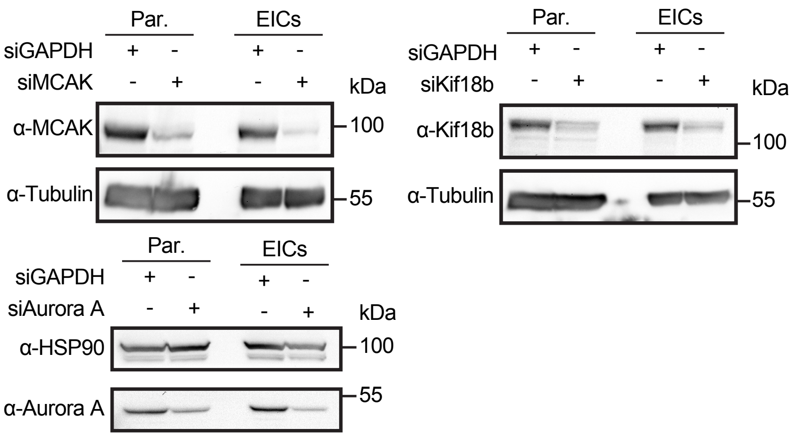

Supplementary Figure 3. Knockdown confirmation of the siRNAs used in this study. Parental and EICs cells were transfected with the indicated siRNAs. 48 hr after transfection, the cells were harvested and protein levels were analyzed by western blot.
