# Supplementary material for: Aurora A, MCAK, and Kif18b promote Eg5-independent spindle formation
Source: Chromosoma. 2016 Jun 29;126(4):473–86. doi: 10.1007/s00412-016-0607-4 (PMC5509784; doi:10.1007/s00412-016-0607-4)
Supplement: Supplementary file 6 — (PDF 1401 kb) [file 412_2016_607_MOESM6_ESM.pdf]

Supplementary Figure 4. Eg5 and Aurora A cooperate to promote bipolar spindle formation.

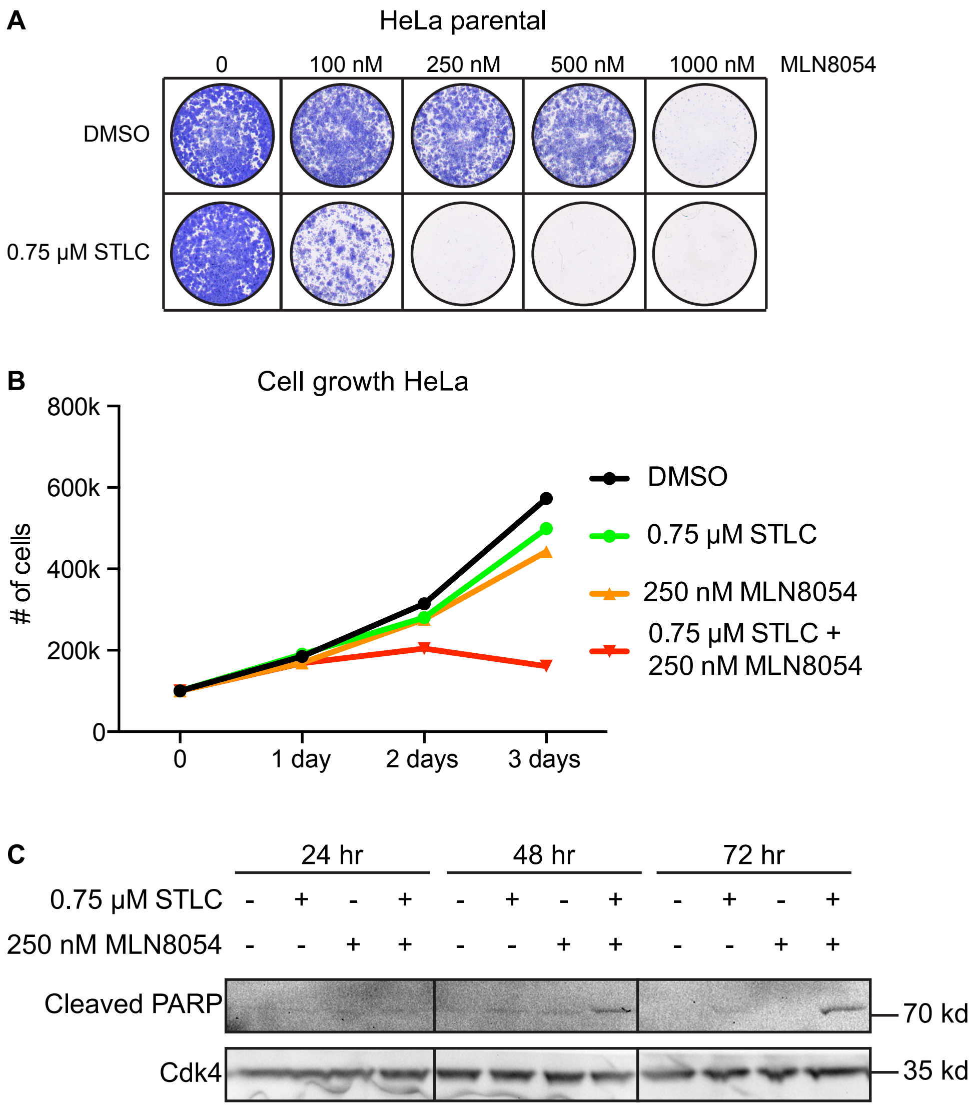

Supplementary Figure 4. Eg5 and Aurora A cooperate to promote bipolar spindle formation. (A) Colony formation assay of HeLa cells. The cells were treated for 5 days with the indicated drug combinations, fixed with methanol and stained with crystal violet. (B) Cell growth assay of HeLa cells. treated with the indicated drug combinations. (C) Western blot analysis showing the level of cleaved PARP as a marker for apoptosis upon 24, 48 and 72 hrs of treatment with the indicated drug combinations. Cdk4 was used as a loading control.
