# Supplementary material for: Aurora A, MCAK, and Kif18b promote Eg5-independent spindle formation
Source: Chromosoma. 2016 Jun 29;126(4):473–86. doi: 10.1007/s00412-016-0607-4 (PMC5509784; doi:10.1007/s00412-016-0607-4)
Supplement: Supplementary file 7 — (PDF 232 kb) [file 412_2016_607_MOESM7_ESM.pdf]

Supplementary Figure 5. Astral MT deform the cortical membrane after reducing cortical tension.

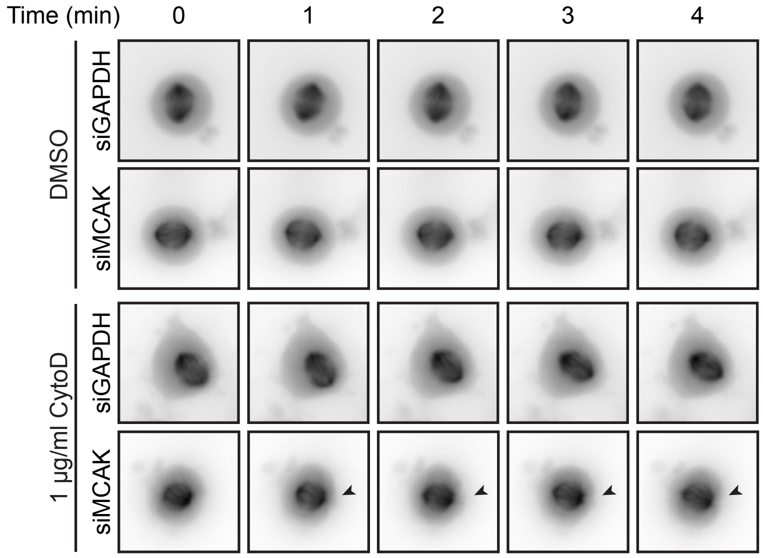

Supplementary Figure 5. Astral MT deform the cortical membrane after reducing cortical tension. Representative stills of HeLa cells expressing GFP-α-tubulin, treated with the indicated siRNAs
Cells were arrested in metaphase using MG132 and treated with DMSO or cytochalasin D.
The black arrow indicates the deformation of the cortex by astral MTs in MCAK-depleted cells treated with cytochalasin D. See also supplementary movies S1-S4.
