# Supplementary material for: Aurora A, MCAK, and Kif18b promote Eg5-independent spindle formation
Source: Chromosoma. 2016 Jun 29;126(4):473–86. doi: 10.1007/s00412-016-0607-4 (PMC5509784; doi:10.1007/s00412-016-0607-4)
Supplement: Supplementary file 8 — (PDF 382 kb) [file 412_2016_607_MOESM8_ESM.pdf]

Supplementary Figure 6. Excessive astral MT nucleation blocks centrosome separation and bipolar spindle formation.

A

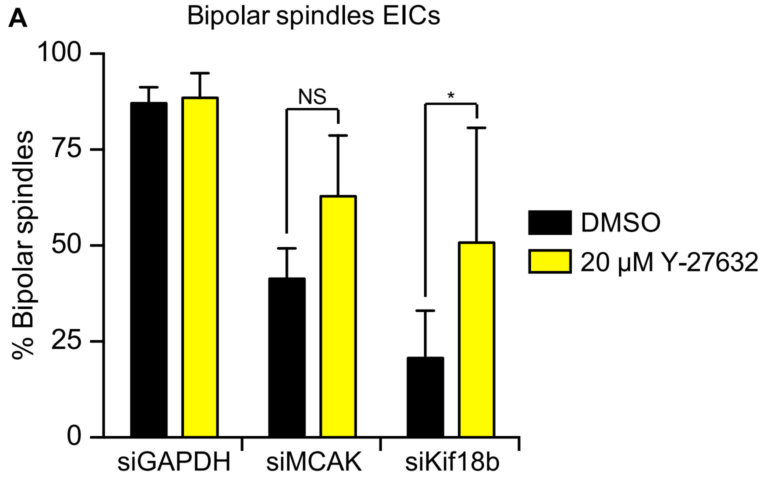

Supplementary Figure 6. Excessive astral MT nucleation blocks centrosome separation and bipolar spindle formation.
(A) Quantification of the percentage of bipolar spindles in EICs. The cells were treated with the indicated siRNAs for 48 hrs. DNA was stained using 4μM SiR-DNA and cells were treated with either DMSO or the Rho kinase inhibitor Y-27632. Results are averages of four independent experiments (n = > 85 cells per condition). *, P <0.01; ns, not significant. p values calculated using two-way ANOVA. Error bars represent s.d.
