# Supplementary material for: Aurora A, MCAK, and Kif18b promote Eg5-independent spindle formation
Source: Chromosoma. 2016 Jun 29;126(4):473–86. doi: 10.1007/s00412-016-0607-4 (PMC5509784; doi:10.1007/s00412-016-0607-4)
Supplement: Supplementary file 9 — (PDF 1115 kb) [file 412_2016_607_MOESM9_ESM.pdf]

Supplementary Figure 7. In vitro kinase assay Aurora A and Kif15

A

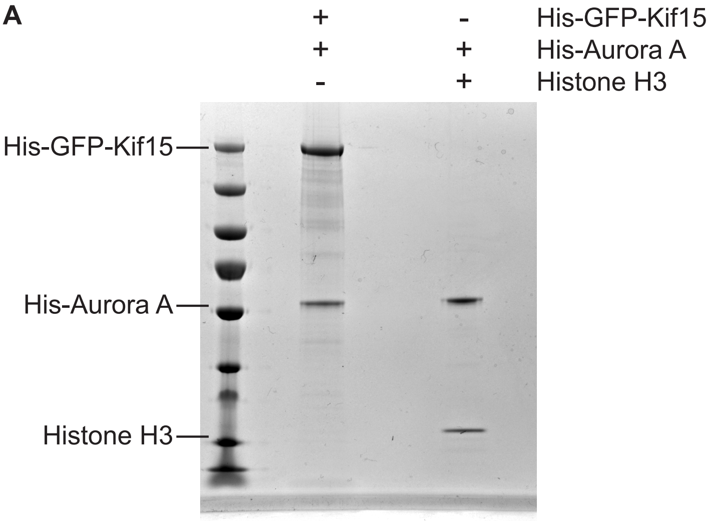

B

Position	Target	Highest PTM score	Sequence motif	Aurora A consensus	In vivo confirmation (reference)
568	S	100%	KLGQGFsPKALKE	No	Dephoure et al., 2008; Olsen et al., 2010; Kettenbach et al., 2011
1134	S	53.9%	LEHVMGsATEYPQ	No	Kettenbach et al., 2011
1141	S	100%	ATEYPQsPKTPPH	No	Olsen et al., 2010; Kettenbach et al., 2011
1144	T	100%	YPQSPKtPPHFQA	No	Olsen et al., 2010; Kettenbach et al., 2011
1169	S	100%	IEDGRAsKTSLQH	Yes	Olsen et al., 2010; Kettenbach et al., 2011
1171	T	92.9%	DGRASKtSLQHLV	No	Kettenbach et al., 2011
1172	S	7.1%	GRASKTsLQHLVT	No	Kettenbach et al., 2011

Supplementary Figure 7. In vitro kinase assay with Aurora A and Kif15. (A) 5 µg recombinant mouse His-GFP-Kif15 was mixed with 0.75 µg human His-Aurora (first lane). Second lane contained 1 µg of Histone 3 and 0.75 µg of human His-Aurora A (control). (B) Phosphorylation sites on Kif15, identified by mass-spectometry. Only sites that were cofirmed previously in vivo are shown. Note that only S1169 and S1172 matched the Aurora A consensus site R-X-[S/T].
