# Supplementary material for: Aurora A, MCAK, and Kif18b promote Eg5-independent spindle formation
Source: Chromosoma. 2016 Jun 29;126(4):473–86. doi: 10.1007/s00412-016-0607-4 (PMC5509784; doi:10.1007/s00412-016-0607-4)
Supplement: Supplementary file 10 — (PDF 166 kb) [file 412_2016_607_MOESM10_ESM.pdf]

Supplementary Figure 8. Expression levels of GFP-Kif15 phospho-mutants

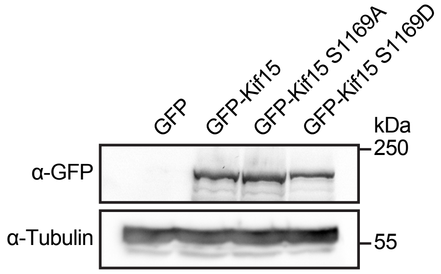

Supplementary Figure 8. Expression levels of GFP-Kif15 phospho-mutants.
U2OS cells were transfected with the indicated constructs. Cells were harvested 48 hours after transfection and protein levels were analyzed by western blot.
